# Development and Pilot Implementation of the Buddy Scheme for New Psychiatry Trainees

**DOI:** 10.1192/bjo.2025.10302

**Published:** 2025-06-20

**Authors:** Naile Aybike Sahin, Elizabeth Keeper

**Affiliations:** MPFT, Stafford, United Kingdom

## Abstract

**Aims:** New psychiatry trainees face several challenges during their initiation to training including adaptation to a new trust, familiarising organisation-specific systems, and understanding training requirements. These challenges affect trainee’s overall confidence and wellbeing. The Buddy Scheme was intended to tackle these by creating a “psychiatry family” for the new trainees. “Psychiatry Family” includes a core trainee and a higher trainee, aiming to foster peer support and mentorship. The goal was to improve new trainee experience, build confidence, and enhance training experience.

**Methods:** In order to identify the need for the Buddy Scheme and determine common challenges faced in the beginning of the training we conducted a semi-structured focus group including 6 trainees from the previous cohort. The discussion was focused on personal experiences, challenges, perspectives and expectations. A thematic analysis was performed to identify common challenges and priorities. According to the findings we designed a guidebook and a checklist to support new trainees, created a semi-structured mentorship schedule and selected mentors. The scheme is currently being piloted, with a preliminary evaluation questionnaire assessing baseline concerns and a post-pilot evaluation planned to measure its impact on trainee confidence, adaptation, and satisfaction.

**Results:** The focus group highlighted several main areas of need, including the need for structured guidance, access to peer/mentor support, and access to relevant resources. Participants expressed enthusiasm for a mentorship programme which can help improve their training experience both professionally and personally. Insights and comments from trainees shaped the structure of tailored resources. The resources include a practical checklist for early requirements, a guidebook containing key trust-specific resources, guidance on Royal College portfolio and compliance expectations in training.

**Conclusion:** Buddy Scheme offers practical and compassionate support to new trainees who are facing many challenges during their transition to their new role. Focus group findings highlight the need for a structured mentorship programme. While Buddy Scheme pilot is yet to be finalized, the initial stages have prepared a strong ground for implementation. Future evaluations will assess the scheme’s impact on trainee adaptation, confidence, wellbeing, and training experience, providing great insights into its potential scalability.

